# A zebrafish model for nevus regeneration

**DOI:** 10.1111/j.1755-148X.2011.00839.x

**Published:** 2011-04

**Authors:** Jennifer Richardson, Zhiqiang Zeng, Craig Ceol, Marina Mione, Ian J Jackson, E Elizabeth Patton

**Affiliations:** 1 Institute of Genetics and Molecular Medicine, MRC Human Genetics Unit, The University of EdinburghEdinburgh, UK; 2 Edinburgh Cancer Research UK Centre, The University of EdinburghEdinburgh, UK; 3 Programs in Molecular Medicine and Cell Dynamics, University of Massachusetts Medical SchoolWorcester, MA, USA; 4 IFOM, the FIRC Institute of Molecular Oncology FoundationVia Adamello, Milan, Italy

Dear Editor,

Nevi are senescent and benign tumors of melanocytes, some of which can progress to melanoma (Gray-Schopfer et al., 2007). BRAF^V600E^ is the most frequent mutation in human nevi and melanoma, and promotes senescence in human melanocytes (Gray-Schopfer et al., 2007). The functional activity of BRAF^V600E^ has been validated in both zebrafish and mouse animal models (Damsky and Bosenberg, 2010; Patton et al., 2010). Both models display nevus-like melanocytic hyperplasia; however, the focus has been on the malignant transformation of these nevi to melanoma and not the nevi themselves.

In zebrafish, the transgenic expression of BRAF^V600E^ from the *mitfa* promoter can promote fish-nevus development, but an additional genetic mutation, for example, in *p53* is required to promote progression to malignancy (Patton et al., 2005). BRAF^V600E^ nevi develop in the young adult fish, and once formed remain static, and do not continue to grow for the remainder of the life of the fish. Thus, like in humans, fish-nevi appear to have limited growth potential, most likely due to oncogenic BRAF-induced senescence pathways. Even in BRAF^V600E^ animals that are deficient for *p53*, only some fish-nevi progress to melanoma (Patton et al., 2005), suggesting that the constraints on fish-nevus growth are robust and that multiple cellular changes are required to promote transformation to melanoma.

We were able to exploit the regenerative capacity of the zebrafish to explore the self-renewal potential of the fish-nevus. The zebrafish pigmentation pattern consists of three pigment cell types: the melanocytes, the iridophores, and the xanthophores (Kelsh et al., 2009). Partial amputation of the fin tissue has previously been studied to dissect the genetic pathways responsible for melanocyte regeneration (Rawls and Johnson, 2000, 2001). Following microinjection of the BRAF^V600E^ transgene into the single-cell embryo (mosaic transgenics), nevi occur randomly, with a proportion in the caudal fin (Appendix S1). Zebrafish fin pigmentation patterns are highly stereotyped, and zebrafish-nevi are clearly distinguishable from normal patterning by ectopic dark, diffusely pigmented and often larger melanocytes. This allowed us to ask whether the constraints on fish-nevus growth are maintained in the context of the regenerating fin tissue. The distal portion of the caudal fin was amputated, removing between one-quarter and one-half of the nevus, and the regrowth of the tail and fish-nevus was recorded. We reasoned that there could be four possible outcomes: nevus regrowth with the regenerating tail fin, enhanced nevus regrowth with the regenerating tail fin, no nevus growth with the regenerating tail fin, or regression of the remaining nevus.

Thirty-four zebrafish displaying fish-nevi within the caudal fin underwent partial amputation (Figure 1B and Table 1). Zebrafish were imaged initially as a reference image, immediately following partial nevus removal (Figure 1A, B) and subsequently at 1-week intervals for at least 3 weeks post-surgery (Figure 1C–G). Four different outcomes were observed (Table 1). The most frequent outcome was complete regrowth of the nevus (regenerate; n = 32; Figure 1C–G). Regenerating fin nevus tissue carried the *mitfa-BRAF*^*V600E*^ transgene cells, as confirmed by genotyping of the regenerate fin tissue (data not shown). However, because the fish are mosaic transgenics, non-nevus tail fin tissue also carried and expressed the transgene preventing us from determining the origin of the repopulating fish-nevus melanocytes. Fin regeneration without regrowth of the nevus was also observed (n = 1; non-regenerate; Figure S1), as was one case of nevus regression in which the remaining segment of nevus appeared to regress leaving the original stripe pattern evident (regression; Figure S2). Rates of fish-nevus regrowth could vary, but all fish-nevi could be clearly seen to begin recurrence within 1 week. Most fish-nevi had recurred by 3 weeks, although one fish required up to 10 weeks for complete regrowth. Three fish showed enhanced regrowth along the length of the tail fin (Figure S3). Thus, we find that most fish-nevi have the potential for recurrence within the context of the regenerating tail fin tissue.

**Table 1:** Summary of BRAF^V600E^ zebrafish nevus response

BRAF^V600E^ nevus response	Number of zebrafish^a^
Recurrent	32
Original size^b^	29
Larger size^c^	3
Nonrecurrence	1
Regression	1

a AB/TE (12 wild-type fish; 2 p53^M214K+/−^), *leopard* (3) or *longfin, leopard* (15), ranging in age from 3 months to 1.5 years.

b Original size recurrent nevi were scored as those nevi that at least regained original nevus size in the regenerating tail fin at 4 weeks. Original size recurrent nevi genotypes: AB/TE (12 wild-type fish; 2 p53^M214K+/−^), *leopard* (3) or *longfin, leopard* (12).

c Larger si2ze recurrent nevi were scored as those nevi that appeared larger at 4 weeks than the original nevus. All larger size recurrent nevi were observed in the *longfin, leopard* line.

**Figure 1:**
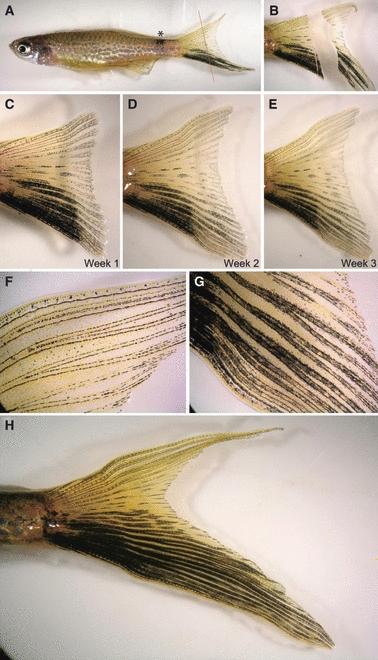
Regeneration of a partially amputated fish-nevus. (A) Mosaic integration of *mitf-BRAF*^*V600E*^ promotes fish-nevus development in wild-type zebrafish. Ectopic melanocytes form a fish-nevus in the tail fin (black tissue, ventral portion of tail fin). The red line indicates the line of amputation; the asterisk indicates an additional fish-nevus on the body. (B) Amputation of a portion of the tail fin, removing the distal portion of the fish-nevus (ventral portion of fin) and normal fin tissue (dorsal portion of fin). The regenerating tail fin was imaged at 1-week intervals following amputation. Regrowth of the tail fin at 1 week (C), 2 weeks (D), and 3 weeks (E). Higher-magnification image of the regenerating tail fin pigmentation in the normal tissue of the dorsal portion of the fin (F) and the regenerating fish-nevus (G). Yellow xanthophores and black melanocytes are clearly visible in the tail fin regenerate. (H) Complete tail fin and fish-nevus regeneration at 4 months.

Fish-nevi recurrence was not altered by different wild-type background (AB or TE strains). We also tested recurrence in zebrafish disrupted for cell-to-cell contact between pigment cells in the mutant line *leopard* (mutation in *connexin 41.8*; (Watanabe et al., 2006) and in zebrafish that have fin overgrowth (*longfin;* mutation in *kcnh2l*; S. Johnson, personal communication). Fish-nevus recurrence was recorded in all fish, regardless of background (Table 1). Notably, fish-nevi could recur after multiple and successive fin amputations (up to at least three times).

We then addressed the cell population that generates the recurrent nevus. One possibility is that the differentiated melanocytes in the remaining fish-nevus undergo migration and/or proliferation to repopulate the regenerating fin. Previous studies have shown that regeneration of the pigment stripes following fin amputation involves unpigmented precursor cells (Lee et al., 2010; Rawls and Johnson, 2000, 2001). Ten fish with regenerative fish-nevi were selected, and recurrence was observed in the presence of N-phenylthiourea (PTU) to block de novo melanin synthesis (Figure 2). In this way, melanin is used as a lineage tracer that pigments the new melanocytes derived from division of original nevus cells or allows for the visualization of migrating nevus cells into the regenerating tail. We found that the tail fins regenerated in PTU lack pigmentation of either the regenerating stripes or nevus cells. Unpigmented cells did repopulate the tail fin; however, because upon the initiation of melanin synthesis (24 h after removal of PTU), we observed pigmented melanocytes in the tail fin and the regenerated fish-nevus. This indicates that the melanocytes responsible for repopulating the fish-nevus, at least in the initial stages, are primarily derived from an unpigmented precursor cell type and not from significant migration or proliferation of differentiated pigmented fish-nevus melanocytes.

**Figure 2:**
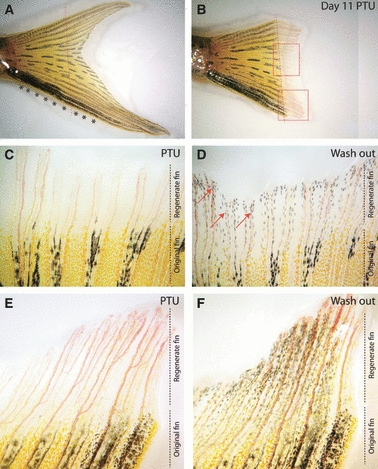
Regenerating fish-nevi develop from an undifferentiated precursor. (A) A zebrafish tail fin with a fish-nevus (asterisks) was partially amputated and regrown in the presence of phenylthiourea (PTU) for 11 days (B). PTU blocks new melanin synthesis (Rawls and Johnson, 2000). Red boxes indicate areas of normal (top) and fish-nevus (bottom) regenerating fin. In the normal and fish-nevus (C and E), the fin regenerates in PTU without pigmentation and the blood vessels are clearly visible. (D and F) After 24 h in fresh water (PTU washout), the melanocytes are clearly visible (red arrows).

Tail fin regeneration did not promote melanoma in any of the recurring fish-nevi. Notably, two nevi fish had *p53*+/− mutations and also did not develop melanoma (up to 3 months post-caudal fin amputation) at the regenerating nevus, despite developing tumors from additional nevi elsewhere on the body (Table 1). Following this, we wondered whether tail regeneration might stimulate tumor formation in tumor prone BRAF^V600E^*p53* lines in which all melanocytes carry the BRAF^V600E^ transgene. We repeated our tail regeneration assay in five stable transgenic BRAF^V600E/V600E^*p53*−/− zebrafish and found no progression to melanoma at the tail fin (followed up to 4 weeks post-amputation; data not shown). Likewise, amputation of the tail fins in the highly tumor prone RAS^V12^ stable lines (Santoriello et al., 2010) also did not stimulate tumorigenesis (followed up to 3 weeks post-amputation; n = 24; data not shown). Thus, in the proliferative environment of the regenerating tail fin, sufficient cellular controls are maintained to prevent tumorigenesis in BRAF^V600E^- and RAS^V12^-expressing melanocytes.

In conclusion, otherwise growth-restricted zebrafish fish-nevi have the potential to repopulate large portions of a nevus from an unpigmented precursor cell type, without promoting tumorigenicity. UV light exposure and BRAF mutations contribute to nevus initiation in humans but the maintenance, recurrence, and regression of nevi are not well understood. Nevi often regress in older people (Tucker et al., 2002), and a proportion of patients display nevi recurrence following removal by surgical curettage or dermabrasion (King et al., 2009). These recurrent nevi are not tumorigenic but can often resemble a dysplastic nevus or melanoma (pseudomelanoma). The source of melanocytes that repopulate a recurrent nevus is unknown, but it has been postulated that the melanocytes may be derived from nearby melanocyte stem cells or residual nevus melanocytes at the site of removal (King et al., 2009). In our model, the regenerative nevi appear to be derived from an unpigmented precursor population, at least during the first 11 days of regeneration. The potential for differing regenerative outcomes of the fish-nevi suggests that fish-nevi may actively regulate and sustain their growth. While we do not know how human nevus maintenance compares with the zebrafish-observed nevus outcomes, this model provides a novel platform to study fundamental questions about nevus maintenance, regrowth, and regression.

## Supporting information

**Figure S1.** A nonrecurrent fish-nevus.

**Figure S2.** A regressive fish-nevus.

**Figure S3.** A recurrent fish-nevus with enhanced regrowth.
