# Supplementary figures and images for: A zebrafish model for nevus regeneration
Source: Pigment Cell Melanoma Res. 2011 Apr;24(2):378–81. doi: 10.1111/j.1755-148X.2011.00839.x (PMC3084990; doi:10.1111/j.1755-148X.2011.00839.x)

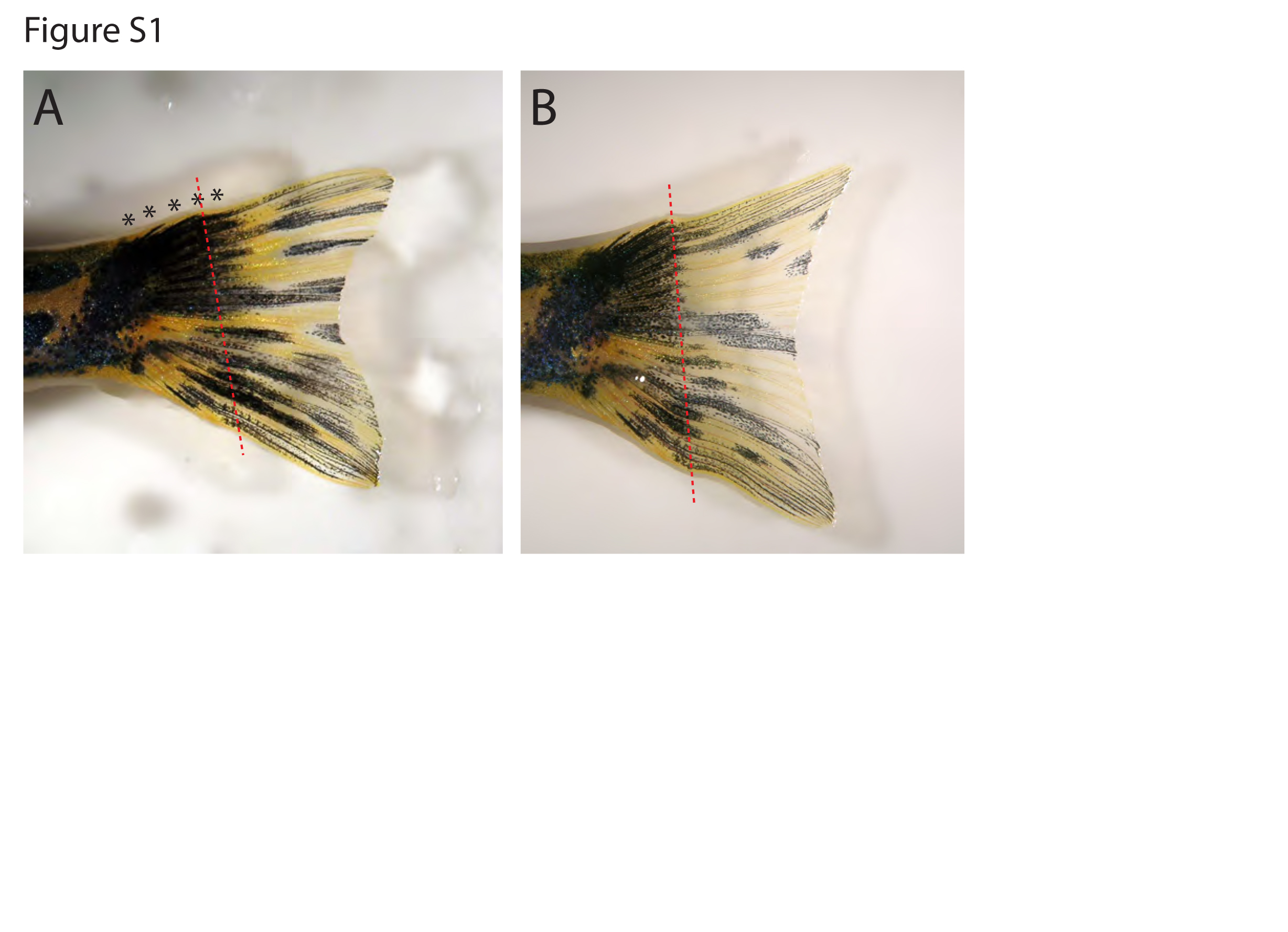

Supplement: Supplementary file 1 [file pcmr0024-0378-SD1.jpg]

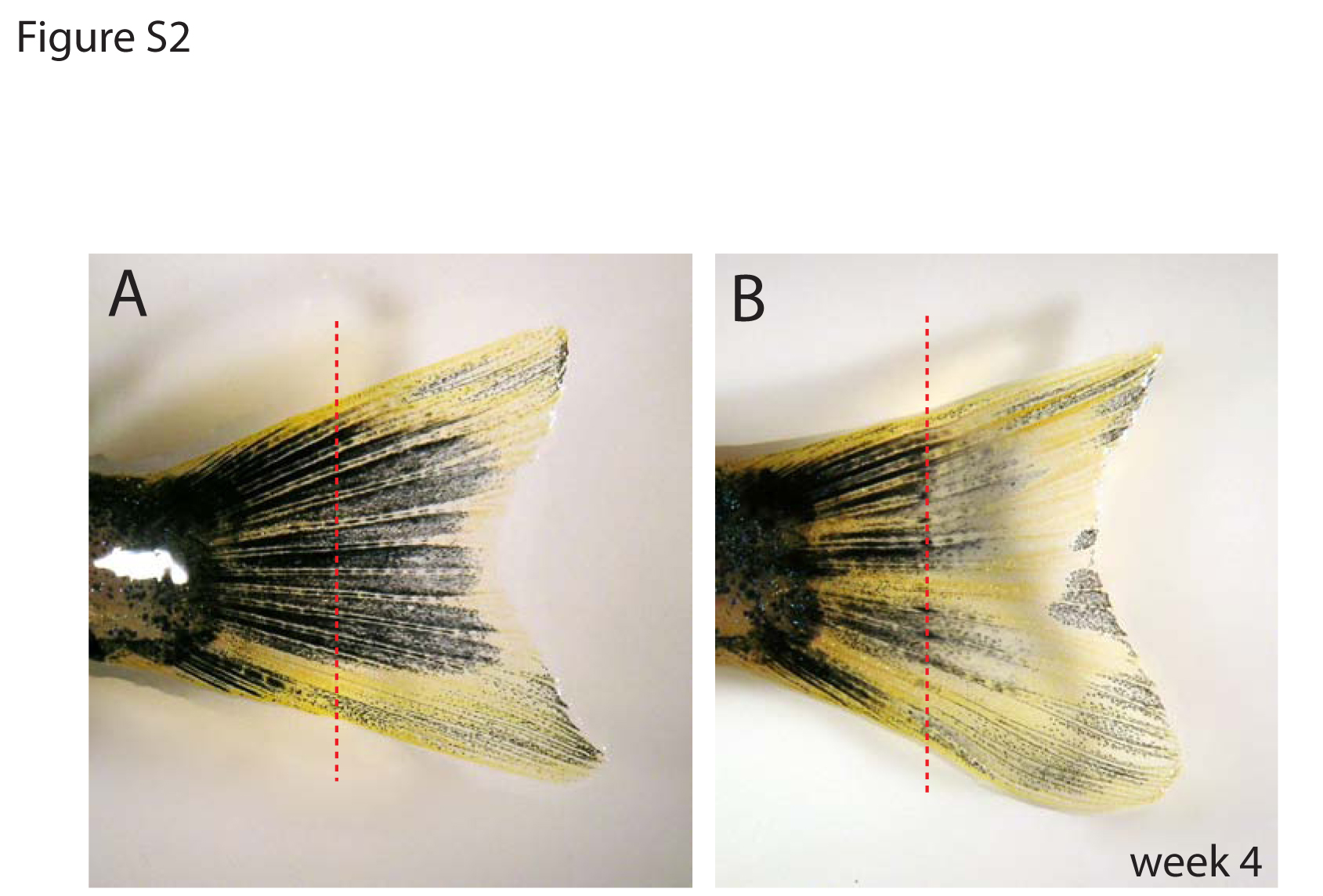

Supplement: Supplementary file 2 [file pcmr0024-0378-SD2.jpg]

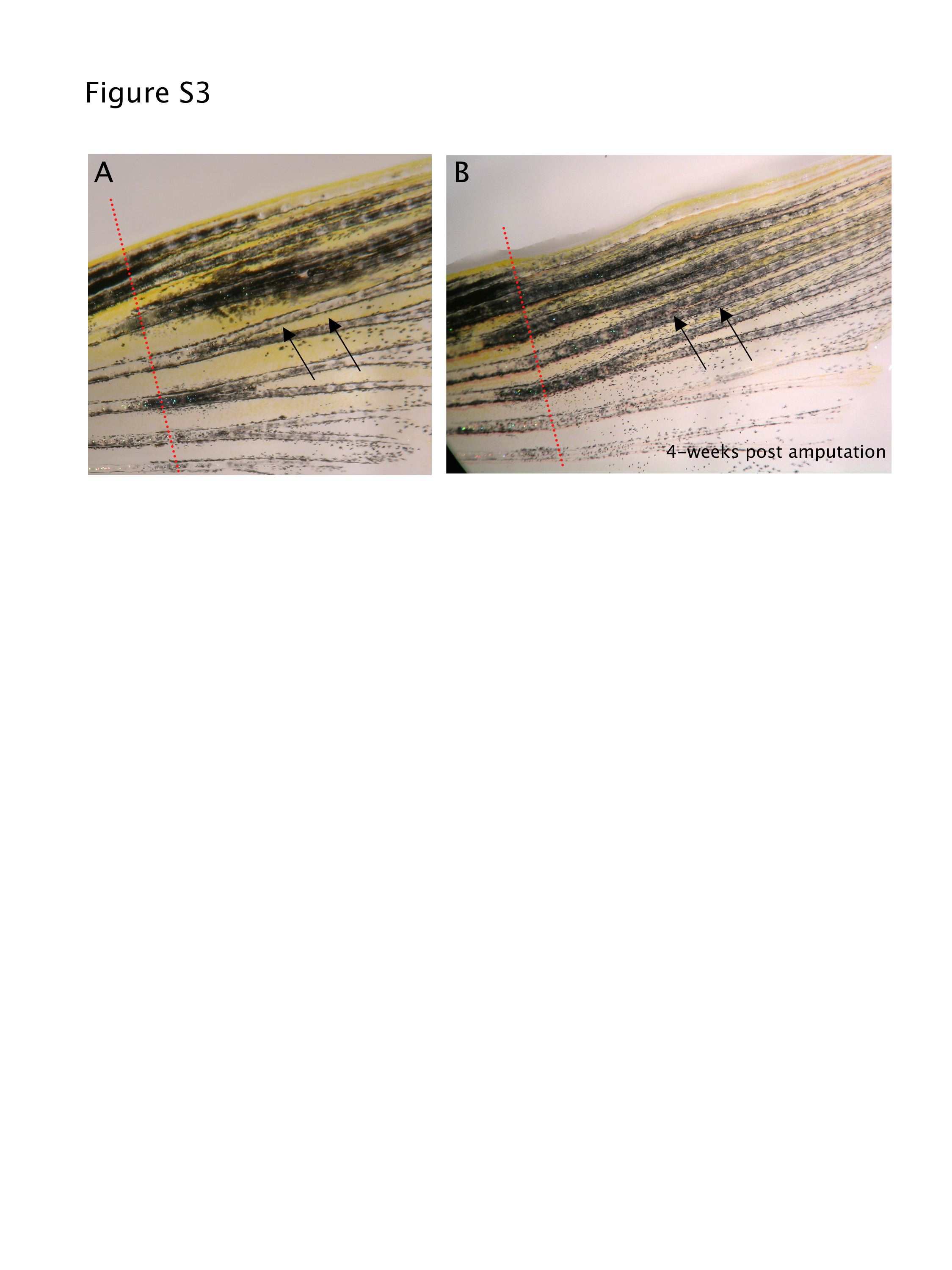

Supplement: Supplementary file 3 [file pcmr0024-0378-SD3.jpg]
