# Supplementary material for: A zebrafish model for nevus regeneration
Source: Pigment Cell Melanoma Res. 2011 Apr;24(2):378–81. doi: 10.1111/j.1755-148X.2011.00839.x (PMC3084990; doi:10.1111/j.1755-148X.2011.00839.x)
Supplement: Supplementary file 4 [file pcmr0024-0378-SD4.doc]

**Appendix S1.** Materials and Methods

**Generation of Mosaic BRAF^V600E^ Zebrafish**

Wild-type (AB, TE, TPL) and mutant zebrafish lines were maintained on a 12hr:12hr light/dark cycle, and were kept at a constant temperature of 28.5˚C. Wild type and mutant lines were set up in breeding pairs the evening before injection. Fish were allowed to breed at the start of the light cycle, and eggs collected immediately following breeding. Gateway™ technology was employed to clone BRAF^V600E^ under the control of the *mitfa* promoter. Single site Gateway™ cloning was carried out between a pENTR™3C entry clone containing human BRAF^V600E^ and a destination vector pT2Kmin-NP Dest RfA carrying and the *mitfa* promoter, flanked by Tol2 transposon sequences (kindly donated by Dr. James Lister, USA). A single LR recombination reaction was carried out between these two vectors using an LR Clonase™ Mix (Invitrogen), according to the manufacturer’s protocol. This construct was co-injected with transposase RNA (both at a concentration of 25ng/µl) directly into the single-celled embryo. Embryos were transferred to the aquarium nursery at 5 days post fertilization and raised to adulthood.

**Caudal fin amputation and Imaging**

Zebrafish were anaesthestised using tricaine methanesulfonate (Sigma). Zebrafish were imaged before amputation for an initial reference image. The distal portion of the caudal fin was removed using a sterile blade, and the fish imaged again, before being returned to the tank to recover. Zebrafish were imaged at the specified time points for each experiment, utilizing the same anesthesia method as for caudal fin amputation.

**PTU treatment**

Following caudal fin amputation, fish were immersed in 30mg/L N-phenylthiourea (PTU; Sigma) dissolved in aquarium system water. The fish were placed into fresh solution of PTU every two days. This was repeated until day 11, at which point the fish were placed into fresh aquarium system water, and monitored for an additional week.

**Genotyping recurrent nevus tissue**

Clipped tail fin and nevi tissues were ground with a pestle in 100 µl of DNA extraction buffer (10 mM Tris pH 8.2, 10 mM EDTA, 200 mM NaCl,0.5% SDS,200 µg/ml proteinase K) in 1.5 ml tubes, then incubated at 56^o^C for 3 hours. The lysate was mixed with 2 volumes of ethanol and 1/20 volume of NaAc (pH5.2) and kept at room temperature for 15 min allowing the DNA to precipitate. The mixtures were centrifuged at 13000rpm for 10 min. DNA pellets were washed in 70% ethanol and dried briefly. DNA samples were finally dissolved in 50 µl of TE buffer (10mM Tris-HCl pH 8.0, 1mM EDTA pH 8.0). One µl of template DNA was used in a 25 µl of PCR reaction. Forward and reverse primers are located in the *mitf* promoter and *BRAF* coding sequence respectively Fwd: GAG GCT TTT GTC GAA TCG GAC CGG TG; Rev: TTG AAC AGA GCC TGG CCC GGC T. PCR conditions are 95^o^C, 3 min for initial denature, then 35 thermocycles ( 95^o^C, 20 sec denature, 56^o^C, 30 sec for annealing and 72^o^C, 60 sec for extension), 72^o^C, 5 min for the last extension. Six µl of PCR product was visualized on 1.0% agarose gel.
